# Prediction of activity spectra for substances

**DOI:** 10.4103/0976-500X.77119

**Published:** 2011

**Authors:** S. Parasuraman

**Affiliations:** *Department of Pharmacology, Jawaharlal Institute of Postgraduate Medical Education & Research, Puducherry, India*

The biological activity spectrum (BAS) is an intrinsic property of a compound that is representative of different pharmacological effects, physiological and biochemical mechanisms of action and specific toxicity (mutagenicity, carcinogenicity, teratogenicity, and embryotoxicity). The activity is largely dependent on the structural nature of a compound.

Biologically active substances have therapeutic and supplementary actions, the latter manifesting as side effects. Some of the major biological activities of a compound become evident during the initial preclinical studies, others during clinical trains and the rest come to light during the postmarketing phase. These newer activities of the compound provide insight for therapeutic applications.

Prediction of activity spectra for substances (PASS) is hosted by the V. N. Orechovich Institute of Biomedical Chemistry under the aegis of the Russian Foundation of Basic Research. The web-based application predicts the biological activity spectrum of a compound based on its structure. It works on the principle that the biological activity of a compound equates to its structure. PASS prediction tools are constructed using 20000 principal compounds from MDDR database (produced by Accelrys and Prous Science). The database contains over 180000 biologically relevant compounds and is constantly updated.

PASS web tool has the ability to predict 3678 pharmacological effects; mechanisms and special toxicities of the molecule including mutagenicity, carcinogenicity, teratogenicity, and embryotoxicity. The predicted activity spectrum includes 65 of 374 pharmacological effects, 176 of 2755 molecular mechanisms, 7 of 50 toxic effects, 11 of 121 metabolism terms at default Pa > Pi cutting points. The web tool can freely display the predicted activity of a molecule at various threshold levels. The PASS training set which has been compiled from various sources including publications, patents, chemical databases, and “gray” literatures consists of over 26000 biological compounds and includes drugs, lead compounds, drug-candidates, and toxic substances.

## WEB PAGE NAVIGATION INFORMATION

The search word “PASS prediction” in various web browsers allows to access directly to the website “http://195.178.207.233/PASS/index.html.” After a hassle free registration the account is activated through an email. After logging-in the web page goes on to the prediction page. The molecule structure of any compound can be attached as a *.MOL file or can be drawn on web site itself using free space given in the website.

## HOW TO DRAW A STRUCTURE OF A MOLECULE

PASS tool will interpret the biological active spectra using 2D structure of molecules. The structure of a molecule may be drawn using ACD/ChemSketch (http://www.acdlabs.com/home/) version 12. The structure of the molecule may be saved ChemSketch 2.0 document (*.SK2) or MDL Mol files (*.mol) and directly uploaded in the PASS prediction website to predict the biological activity spectra of the molecule. The structure can also be drawn directly in PASS prediction website using a JAVA applet that uses a 2D chemical sketch-drawing program (MarvinSketch).

## PASS PREDICTION APPROACH

Activity of the molecule is predicted by “comparing” the structure of new compound with structure of well-known biological active substrate existing in the database. Algorithm of activity spectrum estimation is based on Bayesian approach. The PASS prediction tool will predict the Pa:Pi (active, inactive ratio) at prediction threshold of Pa > 30%, Pa > 50%, and Pa > 70%. Average accuracy of prediction is about 95% according to leave-one-out cross validation (LOO CV) estimation. Accuracy of PASS prediction depends on comprehensive information about biological activity spectrum for each compound available in PASS training set, and therefore, the estimate of biological activity is more accurate.

## APPLICATIONS

The biological activity spectra for 1000 compounds can be calculated in a very short period of time using a computer with a fast processor. The synthesis of the compound and identification takes some days to years, and screening and optimizing the molecule will take more months or years. The computer aided-drug designing will help to optimize the molecules and drug leads and will speed-up the drug development process.

1. Revealing newer mechanism and effects of existing molecules. E.g.: Cavinton (Vinpocetin: alkaloid derivative vinpocetine).
2. To find out most probable new leads. E.g.: Angiotensin converting enzyme inhibitor + Endothelin antagonist.
3. High throughput screening of a series of compounds.
4. Screening of compounds. e.g.: prediction of biological active spectra of molecules.

## LIMITATIONS

1. Prediction of biological activity spectra is based on 2D structure of a molecule and the calculation is not conclusive regarding the activity of a molecule.
2. Prediction does not calculate the molecular energy levels.
3. Sometimes the predicted biological activity may become invalid during preclinical evaluation of the molecule.

## OTHER RELATED WEB TOOLS

http://www.in-silico.de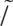 used to calculate the toxic effect of a molecule based on the molecular structure.

http://www.lazar.in-silico.de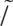 used to calculate the toxic effect of a molecule based on the molecular structure.

www.pharma-algorithms.com – used to calculate the pharmacokinetic profile of a molecule based on the molecular structure.

